# Early Experience With the Iliac Branch Endoprosthesis (IBE) in Managing Iliac Aneurysms

**DOI:** 10.7759/cureus.65915

**Published:** 2024-08-01

**Authors:** Muhammad Numan Zahid, Mohamed S M Elshikhawoda, Sohaib Jararaa, Mahmoud Okaz, Sherif A Mansour, Ebimobo T Keme, Abdillahi Ahmed Roble, Waseem Ahmad, Eyitomi Terry Kenu, Tarig Barakat

**Affiliations:** 1 Vascular and Endovascular Surgery, Glan Clwyd Hospital, Rhyl, GBR; 2 Vascular Surgery, Glan Clwyd Hospital, Rhyl, GBR; 3 Vascular and General Surgery, Glan Clwyd Hospital, Rhyl, GBR; 4 Vascular and General Surgery, Royal Bournemouth Hospital, Bournemouth, GBR; 5 Colorectal Surgery, Royal London Hospital, London, GBR

**Keywords:** endovascular aortic repair (evar), iliac branch endoprosthesis devices, internal iliac artery, common iliac aneurysm, aortoiliac aneurysm

## Abstract

Aim

The objective of this study is to evaluate the feasibility of using iliac branch endoprosthesis (IBE) devices and to examine their short-term outcomes.

Materials and methods

This was a descriptive, retrospective observational study involving 15 patients diagnosed with either aortoiliac or isolated iliac artery aneurysms and treated with an IBE device. Data were collected for patients who received IBE devices at Glan Clwyd Hospital in Rhyl, United Kingdom, from February 2020 to May 2023.

Results

Most patients presented with asymptomatic aneurysms; 86.7% (n = 13) had bilateral common iliac artery (CIA) aneurysms. The mean diameter of the CIA was 38.7 ± 8.8 mm, and the mean diameter of the abdominal aortic aneurysm (AAA) was 39.8 ± 23 mm. For the indications of IBE use, 60% (n = 9) of the patients had iliac aneurysms reaching the intervention threshold, 20% (n = 3) had AAA reaching the threshold, and 20% (n = 3) had aortoiliac aneurysms reaching the threshold. The majority of patients underwent bilateral femoral access (86.7%; n = 13), while 13.3% (n = 2) required both femoral and brachial access. Technical success was achieved in all cases. Aside from 20% (n = 3) of cases where the sac size remained static, the majority of patients (80%; n = 12) experienced sac regression. All patients were free from buttock claudication. A type II endoleak was observed in 33.3% (n = 5) of patients. No reinterventions were reported. The mean primary patency was 30.9 ± 0.7 months, and the follow-up period ranged from 12 to 36 months.

Conclusions

IBEs are an effective medical device, demonstrating a high rate of technical success, minimal need for additional procedures, and a low incidence of complications while maintaining a satisfactory rate of primary patency.

## Introduction

Iliac branch devices have broadened the scope of endovascular aneurysm repair (EVAR) for patients with aneurysmal common iliac arteries (CIAs). These devices use bifurcated modular systems with bridging-covered stents to regulate blood flow into the internal iliac arteries (IIAs) [1]. Historically, the IIA was embolized during EVAR deployment, leading to ischemic complications such as buttock claudication and, in some cases, impotence. However, recent studies have demonstrated that effective preservation of the IIA can be safely achieved, thereby minimizing these risks [2-4].

The literature indicates that the presence of a CIA aneurysm alongside an abdominal aortic aneurysm (AAA) complicates the endovascular repair procedure, increasing the risk of rupture and the need for further intervention [5,6]. Additionally, research shows that approximately 20% of AAA cases are accompanied by a concurrent CIA aneurysm [7-9]. Recent advancements in endovascular devices, particularly iliac branch endoprosthesis (IBE) devices, have significantly improved patient outcomes. This study aims to evaluate the feasibility of using IBEs and assess their short-term outcomes.

## Materials and methods

Study design and population

This was a descriptive, retrospective observational study involving 15 patients diagnosed with either aortoiliac or isolated iliac artery aneurysms who were treated with an IBE device.

Data collection

We collected data from patients who received IBE devices at Glan Clwyd Hospital, Rhyl, United Kingdom, between February 2020 and May 2023. The clinical evaluation included factors such as the patient’s age, gender, aneurysm characteristics, smoking history, comorbidities, primary patency, and the need for reintervention.

Cutoff diameter for intervention

Based on the guidelines in place at the time of the study, a CIA diameter greater than 2.5 cm was considered the cutoff for intervention. For the aorta, intervention was indicated if the diameter exceeded 5.5 cm in males and 5 cm in females. The procedure was performed on patients with either both aneurysms meeting the cutoff criteria or if either aneurysms met the threshold individually.

Device instructions for use (IFU)

The minimum diameter of the CIA should be 17 mm to accommodate the 23-mm main trunk in the proximal landing zone. To prevent the main trunk from becoming infolded or compressed, it should not be positioned within narrow stent-graft limbs. Advancing the branch components of the IIA through a narrow segment graft could lead to compression of the IIA or EIA, and thus should be avoided.

Follow-up

The follow-up schedule included assessments at one, 12, 24, 36, and 48 months after the IBE repair to evaluate the status of the sac and any potential complications. Initial assessments were performed using CT, followed by ultrasound for subsequent evaluations.

Data analysis

The data collected for this study were processed using IBM SPSS Statistics for Windows, Version 21.0 (Released 2012; IBM Corp., Armonk, NY, USA) for data entry, cleaning, and analysis. Descriptive statistics were used to present frequency tables with corresponding percentages and means and standard deviations were reported. The Chi-square test was employed for categorical variables. A significance level of 0.05 or less was considered statistically significant, indicating a substantial relationship between the variables.

Definitions

Primary patency refers to the ability to maintain blood flow without the need for additional intervention or signs of diminished flow after the initial treatment. Technical success denotes the effective deployment of the device within the designated landing zone.

## Results

This study included 15 patients, of whom 14 (93.3%) were male and one (6.7%) was female. The mean age was 76.2 ± 7.5 years. Hypertension was the most frequently reported comorbidity, affecting 53.3% of the patients, while other comorbidities were less commonly recorded (Table 1).

**Table 1:** Patient demographics and comorbidities CKD: chronic kidney disease; COPD: chronic obstructive pulmonary disease; PAD: peripheral vascular disease

Parameters		Frequency	Percentage
Gender	Male	14	93.30%
	Female	1	6.70%
Cardiac disease	Yes	7	46.70%
	No	8	53.30%
Hypertension	Yes	8	53.30%
	No	7	46.70%
Diabetes	Yes	2	13.30%
	No	13	86.70%
Malignancy	Yes	6	40%
	No	9	60%
Smoking	Yes	9	60%
	No	6	40%
CKD	Yes	1	6.70%
	No	14	93.30%
COPD	Yes	6	40%
	No	9	60%
PAD	Yes	2	13.30%
	No	13	86.70%
Total		15	100%

Out of the 15 patients, 86.7% (n = 13) had asymptomatic aneurysms, while the remaining patients presented with symptoms such as pain, tenderness on palpation, and embolization. Additionally, 60% (n = 9) had bilateral CIA aneurysms. The mean CIA diameter was 38.7 ± 8.8 mm, and the mean AAA diameter was 39.8 ± 23 mm (according to the previous European Society for Vascular Surgery guidelines). The most commonly diagnosed aneurysm was an aortoiliac aneurysm (80%; n = 12), with 20% (n = 3) having iliac aneurysms. Regarding the indications for IBE use, 60% (n = 9) had iliac aneurysms that reached the intervention threshold, 20% (n = 3) had AAA reaching the threshold, and 20% (n = 3) had aortoiliac aneurysms reaching the threshold (Table 2).

**Table 2:** Indications of intervention AAA: abdominal aortic aneurysm

Indication	Frequency	Percentage
AAA reached the threshold.	3	20%
Iliac aneurysm reached the threshold.	9	60%
Both aortic and iliac aneurysms reached the threshold.	3	20%
Total	15	100%

All patients underwent IBE-EVAR. Most patients, specifically 86.7% (n = 13), had bilateral femoral access, while only 13.3% (n = 2) required both femoral and brachial access. Technical success was achieved in all cases, and no aneurysmal-related deaths were reported. Although 20% (n = 3) of patients had a static sac size, the majority (80%; n = 12) experienced significant sac regression. All patients were free from buttock claudication and other pelvic ischemic complications. A type II endoleak was observed in 33.3% (n = 5) of patients, but no reinterventions were required. The mean primary patency was 30.9 ± 0.7 months. The mean operative time was 102.4 ± 42.8 minutes, while the mean fluoroscopy time was 31 ± 9.9 minutes. The mean follow-up period was 31 ± 0.9 months.

Cross-tabulation analysis revealed no statistically significant relationship between symptomatic presentation, the site involved, and the type of aneurysm with regard to complications, with p-values of 0.095, 0.287, and 0.264, respectively.

## Discussion

Our study concluded that IBEs yield acceptable outcomes in terms of complications, patency, mortality, and reintervention. IBE devices effectively preserve the IIA and prevent ischemic complications in the pelvis. However, they necessitate a suitable landing zone and a healthy artery. Therefore, careful patient selection is essential for the success of this procedure. Current guidelines recommend preserving at least one of the IIAs during EVAR when a CIA aneurysm is present, unless contraindicated by factors such as an aneurysm in the IIA that requires embolization [10]. When anatomical compatibility is confirmed, IBEs are the preferred devices for maintaining flow to the IIA. The authors conclude that IBEs can be successfully deployed with low complication rates during the perioperative phase. Furthermore, they found that IBEs exhibited satisfactory device integrity and patency rates, consistent with previous studies [11,12], which aligns with our findings.

The device’s IFU specify that a healthy IIA main trunk and the absence of an aneurysm are required. Failure to meet these criteria could jeopardize the landing area and potentially result in adverse outcomes. However, some authors have documented using more distalization to achieve a healthy landing zone rather than opting for embolization of the IIA. Although they reported satisfactory technical outcomes, there was a significant incidence of secondary interventions and endoleak [13-16].

In our investigation, we observed a type 2 endoleak in five patients. However, there were no instances of reintervention, death, or buttock claudication, and we achieved 100% technical success. D'Oria et al., in their study of 74 patients, reported a low incidence of endoleak and reintervention, with only two cases of buttock claudication and no mortality. This discrepancy may be attributed to differences in case numbers and the complexity of the aortic surgeries performed, which were more complex in their study compared to ours [17]. Fargion et al., in their review of IBE outcomes from the pELVIS Registry, which included 157 patients, reported nearly 100% technical success. However, they also encountered type I and III endoleaks that required reintervention and noted perioperative mortality related to patient frailty, prolonged operative time, and high contrast dose. This variation is likely due to differences in the patient age group and the number of cases studied [18].

A recent systematic review and meta-analysis concluded that the use of IBEs to maintain pelvic perfusion is an effective and safe intervention, demonstrating high technical success, low reintervention rates, minimal pelvic complications, and satisfactory patency rates. These findings are consistent with our results [19]. In a multicentric prospective study, the authors concluded that IBE demonstrated satisfactory results regarding primary patency, with a significant reduction in diameters, minimal need for secondary intervention, and adequate clinical outcomes. These findings align with our results [20].

The use of the IBE device for treating isolated IIA aneurysms is not covered by the approved IFU. Nonetheless, there are a few documented case reports on this application [21-23]. D'Oria et al. utilized IBEs for isolated IIA aneurysms and observed either a significant reduction in sac size or a stable sac size [24]. On the other hand, Bahroloomi et al. found that IIA embolization with external iliac artery extension was more effective than IBE in reducing the size of an aneurysmal sac. However, the study also reported occurrences of type 1b endoleak as well as higher rates of limb complications and reinterventions in these patients [25].

This study has several limitations. The data were retrospectively collected, and the study was conducted at a single center. Additionally, the sample size was limited, and the follow-up period was relatively short.

## Conclusions

Our study demonstrated that IBEs are effective medical devices, showing a high rate of technical success, minimal need for additional procedures, and a low incidence of complications while maintaining a satisfactory rate of primary patency. IBEs prove to be a feasible technology when used according to the IFU. However, further research is needed to develop definitive guidelines and assess the feasibility and outcomes of using IBEs beyond the current IFU.

The lack of comprehensive guidelines for IBE device use affects their application. To address this, it is crucial to investigate the technology’s functionality and intricacies more thoroughly and establish precise guidelines for its use.
